# Transcriptomics combined with metabolomics unveiled the key genes and metabolites of mycelium growth in *Morchella importuna*

**DOI:** 10.3389/fmicb.2023.1079353

**Published:** 2023-02-01

**Authors:** Tingting Fan, Rui Ren, Shaojun Tang, Yiyun Zhou, Meng Cai, Wenwen Zhao, Yuelin He, Jun Xu

**Affiliations:** ^1^The Laboratory of Forestry Genetics, Central South University of Forestry and Technology, Changsha, China; ^2^The Center of Culture Preservation, Hunan Institute of Microbiology, Changsha, China

**Keywords:** *Morchella importuna*, mycelium growth, transcriptomics, next generation sequence, metabolomics

## Abstract

Morels (*Morchella*) are one of the most popular edible fungi in the world, especially known for their rich nutrition and delicious taste. Earlier research indicates that the production of fruiting bodies can be affected by the growth of mycelium. To investigate the molecular mechanisms underlying mycelium growth in *Morchella importuna*, we performed transcriptome analysis and metabolomics analysis of three growth stages of the hypha of *M. importuna*. As a result, 24 differentially expressed genes, such as transketolase (tktA), glucose-6-phosphate dehydrogenase (G6PDH), fructose-diphosphate aldolase (Fba), and ribose-5-phosphate isomerase (rpiA), as well as 15 differentially accumulated metabolites, including succinate and oxaloacetate, were identified and considered as the key genes and metabolites to mycelium growth in *M. importuna*. In addition, guanosine 3′,5′-cyclic monophosphate (cGMP), guanosine-5′-monophosphate (GMP), and several small peptides were found to differentially accumulate in different growth stages. Furthermore, five pathways, namely, starch and sucrose metabolism, pentose and glucuronate interconversions, fructose and mannose metabolism, tyrosine metabolism, and purine nucleotides, enriched by most DEGs, existed in the three compared groups and were also recognized as important pathways for the development of mycelium in morels. The comprehensive transcriptomics and metabolomics data generated in our study provided valuable information for understanding the mycelium growth of *M. importuna*, and these data also unveiled the key genes, metabolites, and pathways involved in mycelium growth. This research provides a great theoretical basis for the stable production and breeding of morels.

## 1. Introduction

Morels (*Morchella*) is a genus of ascomycete fungi in the Morchellaceae family (Tan et al., 2021a). Morels are one of the most popular edible fungi in the world because of their rich nutrition and delicious taste. *Morchella* is rich in amino acids and flavoring substances; its bioactive substances are important in regulating body immunity; it is anti-fatigue, anti-oxidant, anti-bacterial, anti-tumor, and also has several other medicinal values (Meng et al., 2010; Nitha et al., 2010, 2013; He et al., 2012; Heleno et al., 2013; Liu et al., 2016). As a veritable rare and delicious edible fungus, the study of the domestication and cultivation of *Morchella* has attracted many scientific researchers. Until now, the domestication and cultivation of morels have a history of more than 130 years (Tan, 2016). Nowadays, artificial cultivation has been realized for many morels, such as *M. importuna, M. sextelata, M. eximia, M. exuberans*, and *M. owneri* (Du et al., 2019). Among them, the main cultivars are *M. importuna*, and *M. sextelata*, which belong to the black morels (Elata Clade) (Du, 2019). Despite the great progress made in the cultivation and domestication of *Morchella*, the molecular mechanisms underlying the biological process of life history, growth metabolism, and fruiting body production of *Morchella* are still less known, which may limit the further industrial cultivation of *Morchella* (Zhao et al., 2018).

The production of morels' fruiting body requires nutritional mycelia and specific external conditions so that the vegetative growth can transfer to reproductive growth, and then the fruiting body primordium formation and fruiting body development start (Chen et al., 1997). Thus, understanding the biological process of mycelium growth can shorten the growth cycle of morels and improve economic benefits. Currently, the artificial cultivation technology of morels mainly adopts cultured mycelium as the strain. However, problems such as unclear provenance and weak mycelium culture technology limit the development of the morels production industry. Therefore, it is of great significance to study the molecular mechanism of *Morchella* mycelium growth and development for *Morchella* strain production. Most of the research on *Morchella* mycelium development focused on exploring reasonable culture substrates and the influence of environmental conditions, for example, detecting mycelial growth status and the bioactive substances in mycelia under different temperatures and different salt stress concentrations, measuring the growth rate and colony growth of morels under different culture mediums, and exploring the effects of different carbon sources, nitrogen sources, inorganic salts, microorganisms, and plant growth regulators on the growth of *Morchella* mycelium (Wang et al., 2021; Sun et al., 2022). Tan et al. found that black morels can utilize the organic carbon content decomposed by an exogenous nutrient bag (ENB), which was thereafter rapidly consumed during morel fruiting (Tan et al., 2019). The authors also found that the morels yield was improved by ${\text{NO}}_{3}^{-}$ while inhibited by ${\text{NH}}_{4}^{+}$ (Tan et al., 2021b). In addition, Yu et al. added a low (50 μL/L) and high concentration (200 μL/L) of 1-octen-3-ol to sandy soil and observed that the morel ascocarp yield was improved by 1-octen-3-ol and that it altered the diversity of soil bacterial communities (Yu et al., 2022a). To evaluate the relationship between the microbes and morel production, Yu et al. collected 23 soil samples and sequenced them using ITS and 16S rDNA amplicon sequencing methods. The authors found that some noteworthy bacterial microbes involved in nitrogen fixation and nitrification were identified in soils with high morel yields, such as *Arthrobacter, Bradyhizobium, Devosia, Pseudarthrobacter, Pseudolabrys*, and *Nitrospira* (Yu et al., 2022b). Meanwhile, the soils that were used or not used for *M. sextelata* cultivation for 0, 1, and 2 years were analyzed, and the results showed that continuous cropping may reduce *M. sextelata* production by acidifying the soil and increasing the abundance of pathogenic fungi (Liu et al., 2022). Although these studies have promoted the understanding of the optimal environmental conditions for *Morchella* growth, the molecular mechanism of mycelium growth is still unclear.

With the development of sequencing technology, the wide application of genomics in the field of biology has been greatly promoted. In addition, metabolomics, as a rapidly developing technology in the post-genome era, also provides a powerful tool for studying the molecular mechanism of biological processes in *Morchella importuna* (Cubero-Leon et al., 2014; Liu, 2020). Liu et al. (2019) performed a comprehensive transcriptome analysis of *Morchella importuna* at the stages of vegetative mycelium (VM), initial sclerotium (IS), and mature sclerotium (MS) by deep transcriptional sequencing and *de novo* assembly. The authors showed that the catabolism of carbohydrates occurred mainly in the vegetative mycelium stage, and the anabolism of the energy-rich substances was the main event of both mycelial growth and sclerotial morphogenesis of *M. importuna* (Liu et al., 2019). Meanwhile, transcriptome analysis of *Morchella importuna* at the mycelial and young fruiting body stages by Hao et al. found that the genes encoding CAZymes, mitochondrial proteins, oxidoreductases, and heat shock proteins had higher expression levels in the young fruiting body stage than in the mycelial stage (Hao et al., 2019). The authors also showed that carbohydrate catabolism and energy metabolism were significantly enhanced in the young fruiting body stage. In 2022, Dong et al. performed an untargeted UPLC-Q-TOF-MS-based metabolomics approach to characterize the metabolite profiles of morels samples from different geographical origins in China (Dong et al., 2022). The authors identified significantly different metabolites including lipids, organic acids, amino acids, and ketones, and suspected that these metabolites may serve as molecular markers indicative of specific regions (Dong et al., 2022).

To further improve the molecular mechanism of mycelium growth in *Morchella importuna*, we performed transcriptome sequencing and metabolites detection using next-generation sequencing technology and UPLC-QTOF/MS technology for the three growth stages of hyphal growth (HG), sclerotium development (SD), and sclerotia maturing (SM). According to the experiment data, differentially expressed genes (DEGs) and differentially accumulated metabolites (DAMs) involved in mycelium growth were detected. In addition, the pathways associated with mycelium growth in *Morchella importuna* were analyzed. This study may help us understand the metabolic mechanisms during the mycelium growth of *M. importuna* and provide valuable genetic information for improving productivity in *Morchella importuna* cultivation.

## 2. Materials and methods

### 2.1. Fungal strain and culture conditions

**Figure 1:**
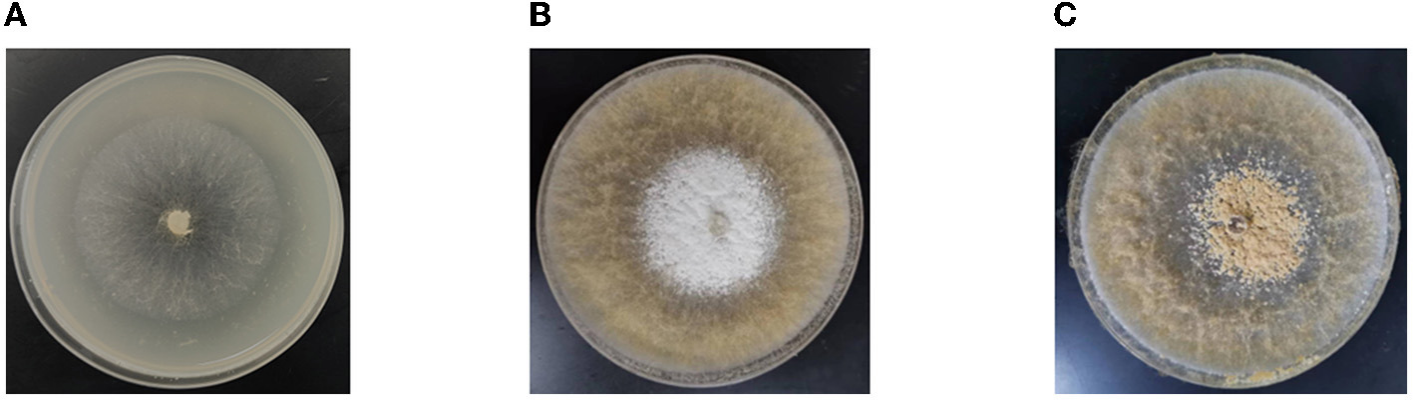
Representative images of different development stages. **(A)** The growth stage of HG. **(B)** The growth stage of SD. **(C)** The growth stage of SM. HG is the period of early hyphae growth. SD is the period of sclerotium development. SM is the period of mature sclerotia.

The *Morchella importuna* strain in this study originates from the greenhouse artificial cultivation of morels in Xiangyin County, Yueyang City, Hunan Province. The strain was preserved in Hunan Agricultural Microbial Germplasm Resource Bank. The *Morchella importuna* strain was grown in a PDA medium (200 g/L potatoes, 20 g/L glucose, 2 g/L KH_2_PO_4_, 2 g/L peptone, 1 g/L MgSO_4_, and 2.2 g/L agar) at 20 °C in the dark. After 2 days, the mycelia began to grow vigorously in the PDA medium and this growth stage was defined as the period of early hyphae growth (HG). Then, the sclerotium developed after 5 days (SD), which further developed into mature sclerotia after 10 days (SM) (Figure 1). The mycelia at the three growth stages were collected with a clean nipper. Three biological repetitions of each stage were performed and a total of nine samples were collected, each containing 0.2 g of mycelia. After drying with filter paper, the samples were frozen immediately in liquid nitrogen and stored at −80°C until RNA extraction and metabolite identification.

### 2.2. RNA extraction and RNA sequencing

Total RNA was extracted by Trizol reagent (Invitrogen, USA) according to the manufacturer's instructions. RNA concentration was determined using Qubit 3.0 (Thermo Scientific, USA), and the quality was assessed using an Agilent 2100 Bioanalyzer (Agilent Technologies, USA). Then, the RNA-seq libraries were prepared using a NEBNext Ultra Directional RNA Library Prep kit (NEB, UK) according to the manufacturer's instructions. Generated libraries were sequenced on a Hiseq 4000 (Illumina, San Diego, USA) platform using a paired-end run (2 × 150 bp). The raw datasets of the nine samples are accessible from the NCBI Sequence Read Archive (SRA) database under the BioProject accession number PRJNA882762.

### 2.3. Data processing and bioinformatics analysis

After sequencing, the raw data were obtained, and then the sequence adaptors and reads with quality under Q20 and reads with lengths less than 50 were removed. The high-quality clean reads were mapped to the *Morchella importuna* reference genome “*Morchella importuna* (assembly ASM344463v2, https://www.ncbi.nlm.nih.gov/assembly/GCF_003444635.1)” using HISAT2 (v2.1.1) (Hierarchical Indexing for Spliced Alignment of Transcripts) software (Kim et al., 2015). Based on the mapping results, ANNOVAR software (Wang et al., 2010) was used to annotate the mapped positions of the reads and to count the distribution of reads in each region of the genome. Transcripts were assembled based on mapping results using StringTie (v1.3.6) software (Pertea et al., 2015). The expression levels of transcripts were evaluated by the RSEM 1.2.31 package using the FPKM (fragments per kilobase of exon per million fragments mapped) method. DEGs between different comparison groups were determined using the R package DESeq2 (Anders and Huber, 2010), and significant DEGs were defined with a threshold of FDR <0.01 and a fold change of ≥2.

The functional annotation of the transcripts was performed by the phyper package in R software. A total of five databases; the Cluster of Orthologous Groups of proteins (COG), the NCBI non-redundant (NR) database, the Swiss-Prot database, the Gene Ontology (GO) database, and the Kyoto Encyclopedia of Genes and Genomes (KEGG) database, were used and 1 × 10^−5^ was used as the E-value threshold.

### 2.4. Extraction and UPLC-QTOF/MS analysis

Each sample was taken out from the −80°C refrigerator and thawed on ice until they could be cut. The thawed sample was homogenized by a grinder (30 Hz) for 20 s. A 400 μL solution (methanol: water = 7:3, v/v) containing internal standard was added to a 20 mg ground sample and shaken at 1,500 rpm for 5 min. After placing on ice for 15 min, the sample was centrifuged at 12,000 rpm for 10 min (4°C). In total 300 μL of supernatant was collected and placed at −20°C for 30 min. The sample was then centrifuged at 12,000 rpm for 3 min (4°C). In 200 μL aliquots of supernatant were transferred for LC-MS analysis. All data were acquired by the LC-MS system following machine orders. The analytical conditions were as follows: UPLC column: waters ACQUITY UPLC HSS T3 C18 (1.8 μm, 2.1 mm^*^100 mm); column temperature: 40 °C; flow rate: 0.4 mL/min; injection volume: 2 μL; solvent system: water (0.1% formic acid): acetonitrile (0.1% formic acid); gradient program: 95:5 v/v at 0 min, 10:90 v/v at 11.0 min, 10:90 v/v at 12.0 min, 95:5 v/v at 12.1 min, and 95:5 v/v at 14.0 min. The mass spectrometry conditions under the positive and negative ionization modes were as follows: voltage: 2.5 kV (ESI+) and 1.5 kV (ESI–); gas flow: 8 L/min (ESI+, ESI–); fragmetor: 135 V (ESI+, ESI–); gas temperature: 325°C (ESI+, ESI–); sheath temperature: 325°C (ESI+, ESI–); sheath flow: 11 L/min (ESI+, ESI–); and nebulizer: 40 V (ESI+, ESI–).

### 2.5. Data processing and metabolite identification

The original data file acquired by LC-MS was converted into mzML format using ProteoWizard software. Peak extraction, peak alignment, and retention time correction were performed by the XCMS program. The “SVR” method was used to correct the peak area. The peaks with a detection rate lower than 50% in each group of samples were discarded. After that, metabolic identification information was obtained by searching the public database.

### 2.6. Data assessment and statistical analysis

Principal component analysis (PCA) was used to observe the population distribution and stability of the whole process of analysis between various samples. The orthogonal partial least squares analysis (OPLS-DA) was used to differentiate the metabolic profile between each sample group and then the differential metabolites were identified. For two-group analysis, differential metabolites were determined by VIP (VIP ≥ 1), *P*-value (*P*-value < 0.05, Student's *t*-test), and absolute Log2FC (|Log2FC| ≥1.0). The PCA and OPLS-DA were performed using the statistics function prcomp within the R software (www.r-project.org).

The hierarchical cluster analysis (HCA) results of samples and metabolites were presented as heatmaps with dendrograms, while Pearson correlation coefficients (PCC) between samples were calculated using the cor function in the R package and presented as only heatmaps. Both HCA and PCC were carried out using the R package ComplexHeatmap. For HCA, normalized signal intensities of metabolites (unit variance scaling) were visualized as a color spectrum.

The Kyoto Encyclopedia of Genes and Genomes (KEGG; www.genome.jp/kegg/) was used for metabolite annotation, biological role explanation, and pathway construction. KEGG pathway analysis of different metabolites was performed using Metabo Analyst 3.0 (www.metaboanalyst.ca/MetaboAnalyst/) (Pang et al., 2020). Significantly enriched pathways were identified with a hypergeometric test's *P*-value for a given list of metabolites.

### 2.7. Quantitative RT-PCR analysis

To validate the reliability of RNA-seq results, we performed quantitative RT-PCR (qRT-PCR). Total RNA was extracted by Trizol reagent (Invitrogen, USA) according to the manufacturer's instructions. The cDNAs were synthesized using a RevertAid First Strand cDNA Synthesis kit (Thermo Scientific, USA) under standard manufacturer instructions. Quantitative RT-PCR analysis was performed using the MonAmp™Fast SYBR^®^ Green qPCR mixture (Monad, CHN) with a QuantStudio™ 5 system (ABI, USA). Three biological replicates were set for each gene. Relative expression levels of each gene were determined *via* the 2^−Δ*ΔCt*^ method (Livak and Schmittgen, 2001). The expression level of the 14-3-3 protein was used as an internal reference gene (Zhang and Liu, 2018). Genes and primers for qRT-PCR analysis are listed in Supplementary Table S1.

## 3. Results

### 3.1. Transcriptome analysis

To explore the molecular mechanism of mycelial growth in *Morchella importuna*, the transcriptomes of three mycelial development stages (HG, SD, and SM) described in the Materials section were compared. In total, nine libraries (three biological replicates were performed for each development stage) were sequenced. After removing the low-quality reads, a total of 53.88 Gb of clean data were generated and each sample was 5.99 Gb on average. The sequence of nucleotide mass fraction Q30 >30 was 92.84% in nine samples, and the GC content was 49.72% (Supplementary Table S2). This result shows that the clean reads had high quality and were suitable for the following analysis. Then, the clean data were mapped to the reference genome and 94.19% of the clean reads could be aligned to the reference genome (Supplementary Table S2).

### 3.2. Analysis of expression levels and the differentially expressed genes

**Figure 2:**
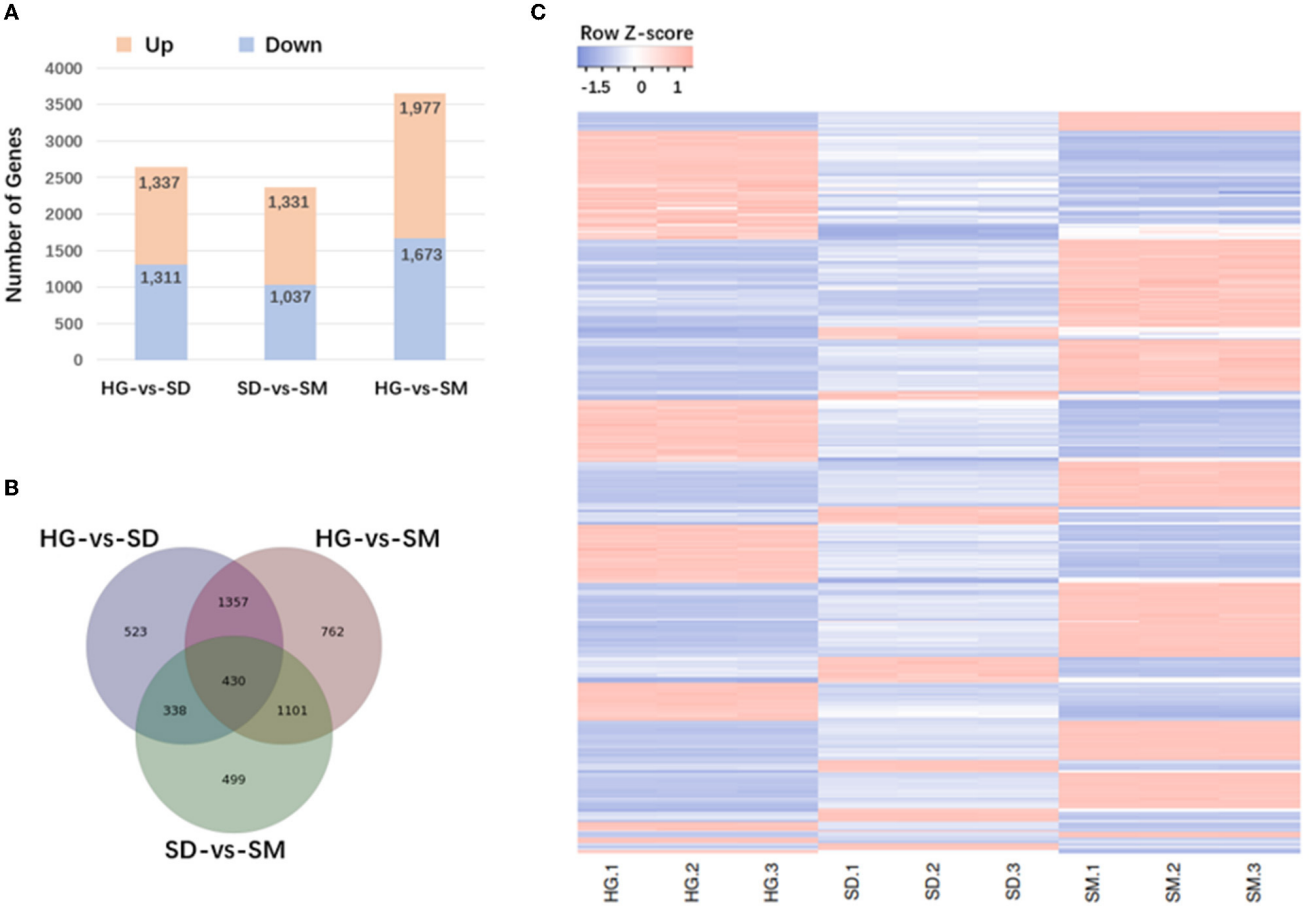
Analysis of differentially expressed genes (DEGs) occurring in the three compared groups. **(A)** Numbers of DEGs in the different comparisons. **(B)** Venn diagram showing the overlapping DEGs in the three compared groups. **(C)** Hierarchical clustering heatmap of 430 DEGs commonly existing in the three compared groups.

The expression levels of transcripts were obtained using the Bowtie 2 program in RSEM. Then, the FPKM (fragments per kb per million reads) values were calculated for the following analysis. The genes differentially expressed at the three mycelial development stages (HG, SD, and SM) were detected. After data filtering, 5,648, 2,368, and 3,650 DEGs were detected in the three compared groups; HG-vs-SD, SD-vs-SM, and HG-vs-SM, respectively (Supplementary Table S3). For the compared group of HG-vs-SD, 1,337 DEGs were upregulated and 1,311 DEGs were downregulated. For the compared group of SD-vs-SM, 1,331 DEGs were upregulated and 1,037 DEGs were downregulated. Finally, in the compared group HG-vs-SM, 1,977 upregulated DEGs and 1,673 downregulated DEGs were obtained (Figure 2A, Supplementary Table S3). Among these DEGs existing in the three compared groups, 430 DEGs commonly existed in the three compared groups (Figure 2B).

To understand the expression pattern of DEGs at the three mycelial development stages in *Morchella importuna*, the hierarchical clustering for DEGs in the three compared groups was conducted (Supplementary Figure S1). The differentially expressed genes in the three groups were mainly classified into high-expression genes (red) and low-expression genes (blue). The DEGs in the three groups fluctuated obviously (upregulated or downregulated) (Supplementary Figure S1). In addition, hierarchical clustering for 430 commonly existing DEGs were also performed. The heatmap showed that these 430 genes fluctuated from stage HG to stage SM (Figure 2C).

### 3.3. Function annotation and enrichment analysis of DEGs

**Figure 3:**
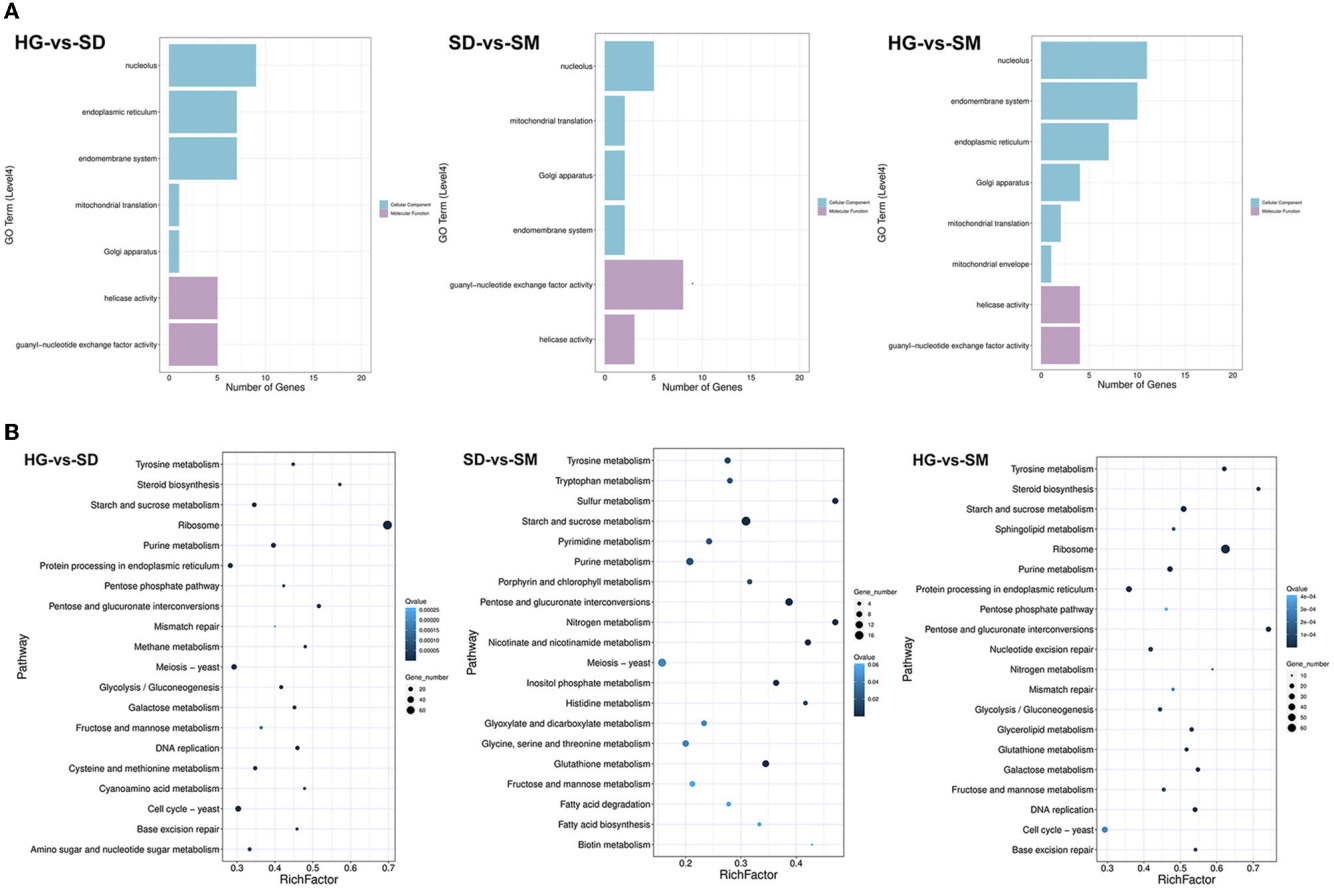
Gene ontology (GO) and KEGG annotation of the DEGs. **(A)** The most enriched GO terms of DEGs in the three compared groups. **(B)** The most enriched KEGG terms of DEGs in the three compared groups.

To investigate the biological processes involved in these DEGs in the three compared groups, Gene Ontology (GO) annotation was performed using DEGs that existed in each compared group. After filtering with the criterion of a corrected *P*-value of ≤ 0.05, the most significant GO term enriched by DEGs existing in the three compared groups were detected (Figure 3A). In the compared group HG-vs-SD, the GO level 4 terms of “nucleolus,” “endoplasmic reticulum,” “endomembrane system,” “mitochondrial translation,” “Golgi apparatus,” “helicase activity,” and “guanyl-nucleotide exchange factor activity” were most significantly enriched. For the compared group SD-vs-SM, the GO level 4 terms of “nucleolus,” “mitochondrial translation,” “Golgi apparatus,” “endomembrane system,” “guanyl-nucleotide exchange factor activity,” and “helicase activity” were the top six most significantly enriched. Meanwhile, the GO level 4 terms of “nucleolus,” “endomembrane system,” “endoplasmic reticulum,” “Golgi apparatus,” “mitochondrial translation,” “mitochondrial envelope,” “helicase activity,” and “guanyl-nucleotide exchange factor activity” were most significantly enriched (Figure 3A).

To further analyze the DEGs-related pathways, KEGG annotation of these transcripts was conducted. Figure 3B shows the top 20 most significantly enriched pathways of DEGs in the three compared groups. Among these, the pathways of “Starch and sucrose metabolism,” “Pentose and glucuronate interconversions,” “Tyrosine metabolism,” “Purine metabolism,” and “Fructose and mannose metabolism” were commonly existing in the three compared groups (Figure 3B).

### 3.4. Overview of metabolomics

To understand the chemical base of *Morchella importuna* at the three mycelial development stages (HG, SD, and SM), metabolomic analyses were performed. In total, 2,100 metabolites were identified in nine samples (three development stages and three biological replicates). Among these, 1,366 metabolites were detected in positive ion mode, and 734 metabolites were detected in negative ion mode (Supplementary Table S4). The composition of metabolites under the positive and negative ions is shown in Supplementary Figure S2. Most of the metabolites were classified as benzene and substituted derivatives (positive ion mode: 17.06%; negative ion mode: 19.07%), followed by the amino acid and its metabolites (positive ion mode: 16.62%; negative ion mode: 8.45%), heterocyclic compounds (positive ion mode: 15.67%; negative ion mode: 13.62%), organic acid and its derivatives (positive ion mode: 10.54%; negative ion mode: 14.03%), and aldehyde, ketones, and esters (positive ion mode: 9.15%; negative ion mode: 7.9%) (Supplementary Figure S2).

**Figure 4:**
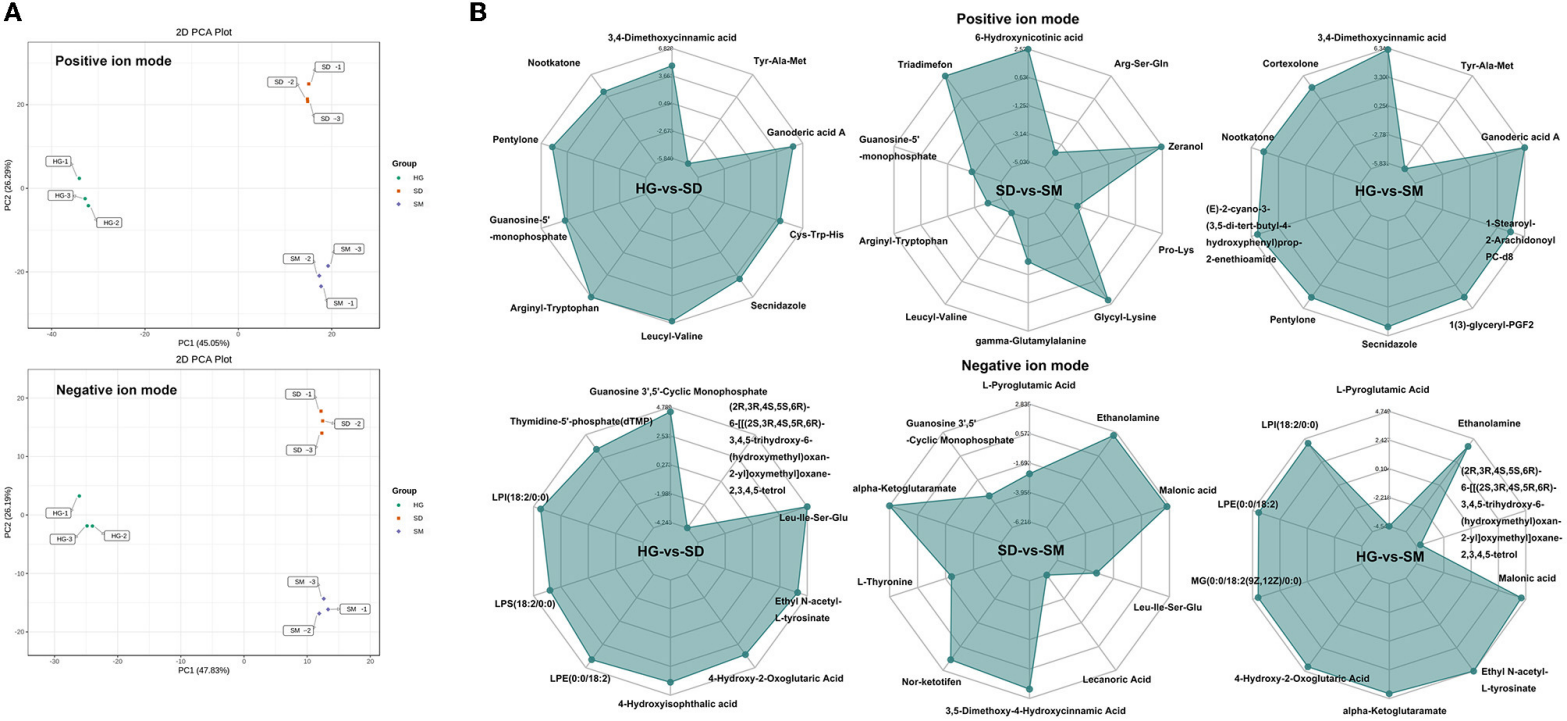
PCA analysis and differential accumulated metabolites (DAMs) analysis. **(A)** PCA analysis of the samples in the three development stages at the positive and negative ion modes. **(B)** Radar chart of significant DAMs in the three compared groups at the positive and negative ion modes.

To assess the difference in total metabolites among each sample, PCA analysis of 1,366 metabolites detected in positive ion mode and 734 metabolites detected in negative ion mode was conducted. The results showed that the first (PC1) and second (PC2) principal components accounted for 39.12 and 22.6%, respectively, for the positive ion mode, and the first (PC1) and second (PC2) principal components accounted for 43.34 and 22.83%, respectively, for the negative ion mode (Figure 4A). The growth development stages of *Morchella importuna* were completely divided into three clusters, revealing that the metabolites in different growth stages were significantly different and metabolomics can be used to study the molecular mechanism of mycelial development.

### 3.5. Analysis of differential accumulated metabolites

According to the criterion mentioned earlier, the 243 (positive ion mode) and 114 (negative ion mode) differential accumulated metabolites were screened out. There were 156, 80, and 185 significantly different metabolites detected in the three compared groups, respectively, under the positive ion mode, and 69, 36, and 93 significantly different metabolites were detected in the three compared groups, respectively, under the negative ion mode (Supplementary Table S5). In order to show the results directly, a hierarchical clustering heat map was plotted based on the FC, *P*-value, and hierarchical clustering analysis (HCA) (Supplementary Figure S3). The heatmap showed that the differential accumulated metabolites separated the two samples in each compared group.

To further investigate the metabolites significantly accumulated in the mycelial development of *Morchella importuna*, the top 10 most accumulated metabolites in each compared group were selected and analyzed (Figure 4B). The annotation of these metabolites showed that these metabolites belong to the metabolite type of amino acid and its metabolites, nucleotide and its metabolites, organic acid and its derivatives, benzene and substituted derivatives, hormones, and hormone-related compounds, ketones, small peptide, heterocyclic compounds, amino acid derivatives, esters, phenolic acids, alcohols, phenolics, and amines (Supplementary material 1). Among these DAMs, five DAMs, namely, guanosine-5′-monophosphate (compound ID: MW0103590), leucyl-valine (compound ID: MW0106515), arginyl-tryptophan (compound ID: MW0105731), guanosine 3′,5′-cyclic monophosphate (compound ID: MEDN0161), and Leu-Ile-Ser-Glu (compound ID: MW0152383), commonly existed in the compared groups HG-vs-SD and SD-vs-SM (Table 1). In these five DAMs, two metabolites (compound IDs: MW0105731 and MW0152383) belong to the metabolite type of amino acid and its metabolites and one metabolite (compound ID: MEDN0161) belongs to the metabolite type of nucleotide and its metabolites, and were upregulated in the development stage of SD and SM as compared to the HG stage (Table 1).

**Table 1:** Five significant DAMs commonly existing in the two or three compared groups.

**Compound ID**	**Compounds**	**Class type**	**Log2FC**		
			**HG-vs-SD**	**SD-vs-SM**	**HG-vs-SM**
MW0103590	Guanosine-5′-monophosphate	Nucleotide and its metabolites	3.93	−2.95	–^*^
MW0106515	Leucyl-Valine	Small peptide	6.57	−5.03	–^*^
MW0105731	Arginyl-tryptophan	Amino acid derivatives	6.83	−4.09	2.74
MEDN0161	Guanosine 3′,5′-cyclic monophosphate	Nucleotide and its metabolites	4.43	−3.22	1.12
MW0152383	Leu-ile-ser-glu	Small peptide	4.79	−3.06	1.73

* − indicates there are no significant differences between the two groups.

### 3.6. Function annotation and enrichment analysis of DAMs

**Figure 5:**
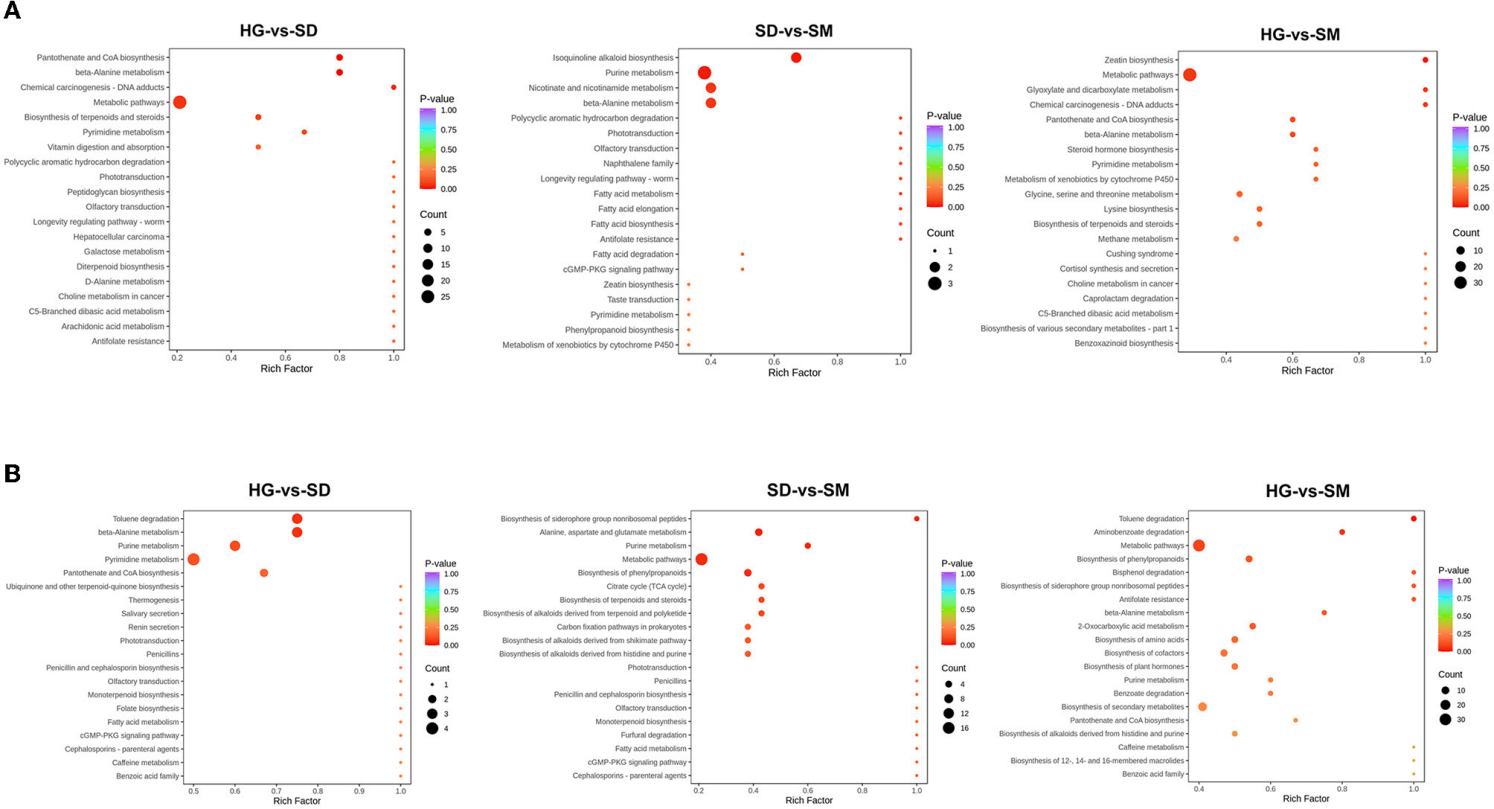
KEGG annotation of DAMs in the three compared groups at the positive and negative ion modes. **(A)** The KEGG annotation of DAMs detected at positive ion modes. **(B)** The KEGG annotation of DAMs detected at negative ion modes.

In organisms, metabolites interact with each other and participate in different metabolic pathways. In order to further understand the metabolic pathways involved in these differentially accumulated metabolites in different development stages, the KEGG database was used to annotate the differentially accumulated metabolites. According to the rich factor and *P*-value, the top 20 most enriched pathways in each compared group were selected and shown in Figure 5. To further investigate the molecular mechanisms of mycelial development, the top 10 most significant pathways in each compared group were analyzed. As a result, the pathways of ko00410 (beta-alanine metabolism), ko01100 (metabolic pathways), and ko01523 (antifolate resistance) commonly existed in the three compared groups. Among these pathways, eight DAMs belong to the metabolites class of organic acid and its derivatives, polyamines, nucleotide and its metabolites, and aldehydes, and amino acids were detected and participated in beta-alanine metabolism (ko00410) (Supplementary material 2). In addition, a total of 84 DAMs were involved in the metabolic pathways (ko01100) and three DAMs were involved in antifolate resistance (ko01523) (Supplementary material 2).

### 3.7. Analysis of the pathways commonly enriched by DEGs and DAMs involved in mycelial growth in *Morchella importuna*

To investigate the molecular mechanisms of mycelium growth in *Morchella importuna*, the significant pathways of DEGs and DAMs commonly enriched were filtered using combined RNA-seq data and metabolomic data. As a result, seven pathways including the pentose phosphate pathway (ko00030), pyruvate metabolism (ko00620), cyanoamino acid metabolism (ko00460), glycine, serine, and threonine metabolism (ko00260), beta-alanine metabolism (ko00410), glycerolipid metabolism (ko00561), and glyoxylate and dicarboxylate metabolism (ko00630) were detected in both DEGs and DAMs participated.

In the pentose phosphate pathway (ko00030), one transketolase (tktA) encoding gene (GeneID: 70146952) was upregulated in the SD and SM stages among the three compared groups, and one differentially accumulated metabolite, glycerate-3P (compound ID: MW0168638), was downregulated in the SD and SM stages in the compared groups HG-vs-SD and HG-vs-SM (Figure 3, Table 2). In addition, the glucose-6-phosphate dehydrogenase (G6PD, GeneID: 70149832), 6-phosphogluconate dehydrogenase decarboxylating (PGD, GeneID: 70142728), fructose-bisphosphate aldolase 1 (FBA, GeneID: 70143593), ribulose-phosphate 3-epimerase (RPE, GeneID: 70146632), ribose 5-phosphate isomerase (rpiA, GeneID: 70148868), phosphoglucose isomerase (GPI, GeneID: 70147960), and phosphoglucomutase (pgm, GeneID: 70143575) encoding genes were upregulated in at least one compared group (Figure 3, Table 2).

**Table 2:** DEGs and DAMs participating in the seven pathways involved in mycelium growth.

**Pathway**	**DEGs/DAMs (GeneID/compoundID)**	**Gene/compound**	**Log2FC**		
			**HG-vs-SD**	**SD-vs-SM**	**HG-vs-SM**
Pentose phosphate	GeneID: 70146952	Transketolase (tktA)	2.76	3.62	6.58
(ko00030)	GeneID: 70149832	Glucose-6-phosphate dehydrogenase (G6PD)	1.44	−0.09	1.37
	GeneID: 70142728	6-phosphogluconate dehydrogenase decarboxylating (PGD)	0.74	2.08	2.87
	GeneID: 70143593	Fructose-bisphosphate aldolase 1 (FBA)	1.01	−0.42	0.62
	GeneID: 70146632	Ribulose-phosphate 3-epimerase (RPE)	1.85	−0.21	1.66
	GeneID: 70148868	Ribose 5-phosphate isomerase (rpiA)	2.10	−0.37	1.76
	GeneID: 70147960	Phosphoglucose isomerase (GPI)	1.80	0.37	2.19
	GeneID: 70143575	Phosphoglucomutase (pgm)	0.93	0.61	1.56
	CompoundID: MW0168638	Glycerate-3P	−1.03	–*	−1.06
Pyruvate metabolism	GeneID: 70148368	Fumarate hydratase encoding gene (fumC)	1.70	1.02	2.75
(ko00620)	GeneID: 70146571	Malate dehydrogenase (MDH2)	1.15	−0.44	0.72
	GeneID: 70147239	Pyruvate carboxylase (PC)	1.34	−0.40	0.97
	GeneID: 70141564	Pyruvate kinase (PK)	1.06	−0.32	0.75
	GeneID: 70144163	Thiolase (atoB)	−1.78	−0.33	−2.09
	CompoundID: MEDN0704	Oxaloacetate	–^*^	−1.24	−1.99
	CompoundID: MW0009773	Succinate	–^*^	−1.38	–^*^
	CompoundID: MW0013541	2-propylmalate	1.30	–^*^	–^*^
Glycerolipid metabolism	GeneID: 70143817	HAD-like protein (GPP1)	−1.42	−1.51	−2.91
(ko00561)	GeneID: 70146115	Dihydroxyacetone kinase (DAK)	1.20	0.32	1.54
Glyoxylate and dicarboxylate	GeneID: 70144131	P-loop containing nucleoside triphosphate hydrolase protein (GLYK)	0.69	0.84	1.55
metabolism (ko00630)	GeneID: 70145180	Citrate synthase (CS)	−0.34	−0.72	−1.04
	CompoundID: MEDL02012	Hydroxypyruvate	–^*^	–^*^	−2.38
	CompoundID: MW0168638	3-phospho-D-glycerate	−1.03	–^*^	−1.06
Glycine, serine and	GeneID: 70148037	FAD dependent oxidoreductase (PIPOX)	0.15	1.73	1.91
threonine metabolism	GeneID: 70143875	PLP-dependent transferase (AGXT)	0.91	0.18	1.11
(ko00260)	GeneID: 70142115	Homoserine dehydrogenase (HD)	1.20	−0.64	0.57
	GeneID: 70148062	Aspartate-semialdehyde dehydrogenase (asd)	1.36	−0.10	1.29
	GeneID: 70141898	Aspartokinase (lysC)	−1.58	−1.14	−2.70
	CompoundID: MEDP0039	Betaine	–^*^	–^*^	1.62
	CompoundID: MW0105618	5-aminolevulinic acid	–^*^	–^*^	−1.83
Cyanoamino acid	GeneID: 70141410	Gamma-glutamyltranspeptidase (GGT1_5)	1.13	−0.17	0.98
metabolism (ko00460)	GeneID: 70141450	Isoaspartyl peptidase (iaaA)	−1.67	0.93	−2.46
	CompoundID: MW0105914	L-aspartate	−1.32	–^*^	−1.07
	CompoundID: MW0105888	L-asparagine	−2.03	–^*^	−2.73
	CompoundID: MEDP2182	3-aminopropiononitrule	2.62	–^*^	–^*^
beta-alanine metabolism	CompoundID: MEDP0178	Uracil	1.52	−1.34	–^*^
(ko00410)	CompoundID: MW0169093	(R)-pantothenate	1.15	–^*^	1.77
	CompoundID: MW0113020	4-aminobutanal	–^*^	−1.17	−1.34
	CompoundID: MEDP1838	Spermine	−1.69	–^*^	−2.24

* − indicates that there are no significant differences between the two groups.

For the pathway of pyruvate metabolism (ko00620), two DAMs, oxaloacetate (compound ID: MEDN0704), and succinate (compound ID: MW0009773), were downregulated in the growth stage of SM while 2-propylmalate (compound ID: MW0013541) was upregulated in the growth stage of SD in the compared group HG-vs-SD (Figure 3, Table 2). This result means that the oxaloacetate and succinate were decreased and the 2-propylmalate increased within the mycelium growth. The fumarate hydratase encoding gene (fumC, GeneID: 70148368) was identified and upregulated in the growth stages of SD and SM in the three compared groups. Malate dehydrogenase (MDH2, GeneID: 70146571), pyruvate carboxylase (PC, GeneID: 70147239), and pyruvate kinase (PK, GeneID: 70141564) were also upregulated within the mycelium growth. The DEG thiolase (atoB, GeneID: 70144163) was downregulated in the growth stages of SD and SM in the three compared groups (Table 2).

Two genes, HAD-like protein (GPP1, GeneID: 70143817) and citrate synthase (CS, GeneID: 70145180), participated in glycerolipid metabolism (ko00561) as well as glyoxylate and dicarboxylate metabolism (ko00630), respectively, and were downregulated within the mycelium growth. Dihydroxyacetone kinase (DAK, GeneID: 70146115) and the P-loop containing nucleoside triphosphate hydrolase protein (GLYK, GeneID: 70144131) were upregulated in the growth stages of SD and SM in the three compared groups (Figure 3, Table 2). In addition, the metabolites of hydroxypyruvate (compound ID: MEDL02012) and 3-phospho-D-glycerate (compound ID: MW0168638) were detected and downregulated in the growth stage of SM compared to the HG stage in this pathway. For the pathways of glycine, serine, and threonine metabolism (ko00260), FAD-dependent oxidoreductase (PIPOX, GeneID: 70148037), PLP-dependent transferase (AGXT, GeneID: 70143875), homoserine dehydrogenase (HD, GeneID: 70142115), aspartate-semialdehyde dehydrogenase (asd, GeneID: 70148062), and Betaine (compound ID: MEDP0039) were identified and upregulated in the growth stages of SD and SM in the compared groups HG-vs-SD and HG-vs-SM. The aspartokinase (lysC, GeneID: 70141898) encoding gene and metabolites of 5-aminolevulinic acid (compound ID: MW0105618) in this pathway were downregulated in the growth stage of SM in the compared group HG-vs-SM (Figure 3, Table 2). For the cyanoamino acid metabolism (ko00460) and beta-alanine metabolism (ko00410), three DAMs; uracil (compound ID: MEDP0178), (R)-pantothenate (compound ID: MW0169093), and 3-aminopropiononitrule (compound ID: MEDP2182) were identified and upregulated in the growth stage of SD in the compared group HG-vs-SD, while three DAMs, L-aspartate, L-asparagine, and spermine, were downregulated in the growth stages of SD and SM in the compared groups HG-vs-SD and HG-vs-SM (Figure 3, Table 2). Meanwhile, two genes participated in cyanoamino acid metabolism, the gamma-glutamyltranspeptidase (GGT1_5, GeneID:70141410) encoding gene was upregulated in the growth stages of SD and SM in the compared groups HG-vs-SD and HG-vs-SM, and the isoaspartyl peptidase (iaaA, GeneID: 70141450) was downregulated in growth stages of SD and SM at compared groups of HG-vs-SD and HG-vs-SM (Figure 3, Table 2).

### 3.8. Dynamic transcripts and metabolites changes during the three development stages of *Morchella importuna*

Based on the expression pattern and clustering method, three subclusters were identified by all DEGs (Supplementary Figure S4). Among them, there were 482, 3,020, and 1,508 genes classified into three subclusters (Supplementary Figure S4, Supplementary material 3). The DEGs in subcluster_1 and subcluster_3 showed similar expression patterns and tended to be highly expressed in SD and remained stable in the SM stage. The expression pattern of DEGs in subcluster_2 showed that the expression was stable in HG and SD stages, and increased in the SM stage. This implied that distinctly expressed modules played their own specific roles during the different growth periods of *Morchella importuna*. Meanwhile, the clustering results indicated that more genes were activated in the SM stage. The 12 DEGs out of 24 potential related genes that participated in ko00030 (pentose phosphate pathway), ko00620 (pyruvate metabolism), ko00561 (glycerolipid metabolism), and ko00630 (glyoxylate and dicarboxylate metabolism), which belong to the carbohydrate metabolism pathway, were classified into subcluster_1 and subcluster_3. This hinted that the carbohydrate metabolism-related genes were activated at the SD stage and afforded more carbohydrates to promote mycelium growth.

In addition, the trend of the relative content of metabolites in different stage samples was obtained through analysis of K-means. The results showed that these DAMs could be classified into 10 different patterns in positive ion mode and eight different patterns in negative ion mode (Supplementary Figure S5, Supplementary material 4). The content changes of DAMs in sub-classes 1, 4, and 5 in positive mode and subclasses 1, 5, and 7 in negative mode showed an up–up pattern, and the levels of these metabolites were increased within the mycelium growth. The levels of agmatine (compound ID: MW0110539) that are associated with arginine metabolism gradually increased with the growth of *Morchella importuna*. Arginine is an essential metabolite in many cell and development processes (Zrenner et al., 2006). The content levels of DAMs in subclasses 2, 3, 8, and 10 in the positive mode and subclasses 4 and 8 showed a decrease in the development stage. The carbohydrate metabolites such as melibiose and the carbohydrate metabolism-related metabolites such as 3-phospho-D-glycerate (compound ID: MW0168638), citric acid (compound ID: MW0106168), and oxalacetic acid (compound ID: MEDN0704) were decreased during the growth of *Morchella importuna*. This means that the carbohydrates were stored during the HG, and the level decreased with the development of *Morchella importuna*. Moreover, the up–down pattern appeared in subclasses 6, 7, and 9 in the positive mode and 2, 3, and 6 in the negative mode. Dihydromonacolin L acid (compound ID: MW0148515), umbelliferone (compound ID: MW0139900), pheophorbide a (compound ID: MW0155495), N-acetyl-5-hydroxytryptamine (compound ID: MW0108605), 4-hydroxybenzoic acid (compound ID: MW0005091), and succinic acid (compound ID: MW0009773), which were involved in the biosynthesis of secondary metabolites were upregulated during the SD stage and downregulated in the SM stage. These accumulated secondary metabolites could mediate ecological interactions and may improve growth viability (Deng et al., 2021).

### 3.9. Validation of candidate genes by qRT-PCR analysis

To validate the reliability of RNA sequencing, eight DEGs containing two genes (PGD- and RPE-encoding gene) involved in the pentose phosphate pathway as well as the GPP1 encoding gene involved in the pathway of glycerolipid metabolism were randomly selected. The results of the qRT-PCR experiment showed that most of these genes had similar expression tendencies to the RNA-sequencing data (Supplementary Figure S6). This result indicated that the RNA-sequencing data were reliable.

## 4. Discussion

Morels are rich in nutrition, contain a variety of active and flavor substances, and are the most famous edible fungus in the world (Sheikh et al., 2015; Sambyal and Singh, 2021). However, the unstable yield of morels has seriously hindered the healthy development of the morels industry. Basic research on the developmental biology of morels is the key to solving the problem of stable production and breeding of morels. Earlier research indicates that the sclerotium is the most important growth stage in morel growth development and is closely related to the production of fruiting bodies (Ower, 1982; Barseghyan et al., 2012). However, the latest experiment showed that there is no inevitable correlation between sclerotium and fruiting bodies' production. Strains with sclerotium may not produce fruiting bodies, and strains with few sclerotia may also produce mushrooms (He et al., 2016; Zhao et al., 2018). In addition, during the cultivation, some strains can produce fruiting bodies when the mycelium is fully grown and sown. The sown time does not wait for the formation of sclerotium. The strain sowed in this way had a short fruiting time and stable yield (Zhao et al., 2018). Therefore, in this study, we focused on the biological process of morel mycelium development and tried to investigate the crucial genes and metabolites involved in mycelium growth using transcriptome and metabolome analyses.

The carbon source was recognized as the major material base of the cytoskeleton and an important nutritional requirement for the growth and development of fungi (Yang et al., 2013). The research found that the mycelial biomass of hairy bracket mushrooms was the highest when sucrose was the carbon source (Yang et al., 2013). In our study, the pathways of “Starch and sucrose metabolism,” “Pentose and glucuronate interconversions,” and “Fructose and mannose metabolism” were the most differentially expressed genes enriched and commonly existed in the three compared groups (Figure 4B). The tyrosine metabolism pathway is the starting point for the production of a variety of structurally diverse natural compounds in plants (Xu et al., 2020). The most DEGs enriched in the pathway of tyrosine metabolism in our study means that a variety of active substances related to tyrosine metabolism are produced to maintain mycelial growth during mycelial development. Similarly, most of the different metabolites that participated in the pathway of tyrosine metabolism were identified by the analysis of primary metabolites of *Morchella* fruit bodies and mycelium (Yang et al., 2021). Purine nucleotides play important roles in nucleic acid constitutes, energy metabolism, signal transduction, and coenzyme factor (Pantazopoulou et al., 2007). Purine metabolism is required for primary metabolism, secondary metabolism, and gene expression and participated in most basic cellular and biochemical processes. It always plays an important role in the growth and development of eukaryotes and prokaryotes. In this study, the pathways of tyrosine metabolism and purine nucleotides also enriched by DEGs existed in the three compared groups (Figure 3B), and we speculated that these pathways are essential for mycelium development in *Morchella importuna*.

As a secondary messenger, guanosine 3′,5′-cyclic monophosphate (cGMP) plays an important role in regulating plant growth and development (Gross and Durner, 2016). A variety of cellular activities are controlled by the signal molecules, such as cyclic nucleotides, guanosine 3′,5′-cyclic monophosphate (cGMP) in plants (Swiezawska-Boniecka et al., 2021). Guanosine-5′-monophosphate (GMP) is the precursor molecule of cGMP, formed by condensation of phosphate and hydroxyl group on the fifth carbon atom of nucleotide ribose, and is one of the five nucleotides that constitute nucleic acid. Among the differentially accumulated metabolites identified in this study, guanosine 3′,5′-cyclic monophosphate (cGMP) and guanosine-5′-monophosphate (GMP) were increased from the HG stage to SD (Table 1), which means plentiful nucleotides associated with mycelium development were accumulated and provided growth sources. Besides the two DAMs mentioned earlier, the small peptide molecular, leucyl-valine, arginyl-tryptophan, and Leu-Ile-Ser-Glu were also identified and significantly differed in the three compared groups (Table 1). Recent studies have found that some long-distance molecular signals, such as small peptides, microRNA, mRNA, second messengers, and proteins, can transmit extracellular stimuli from sensing tissues to target organs, so as to systematically regulate plant development process and environmental response (Takahashi and Shinozaki, 2019). Although there is no research showing that these small peptides detected are directly related to mycelial development, we hypothesized that these small peptides may work with other molecules to regulate the growth and development of mycelia.

**Figure 6:**
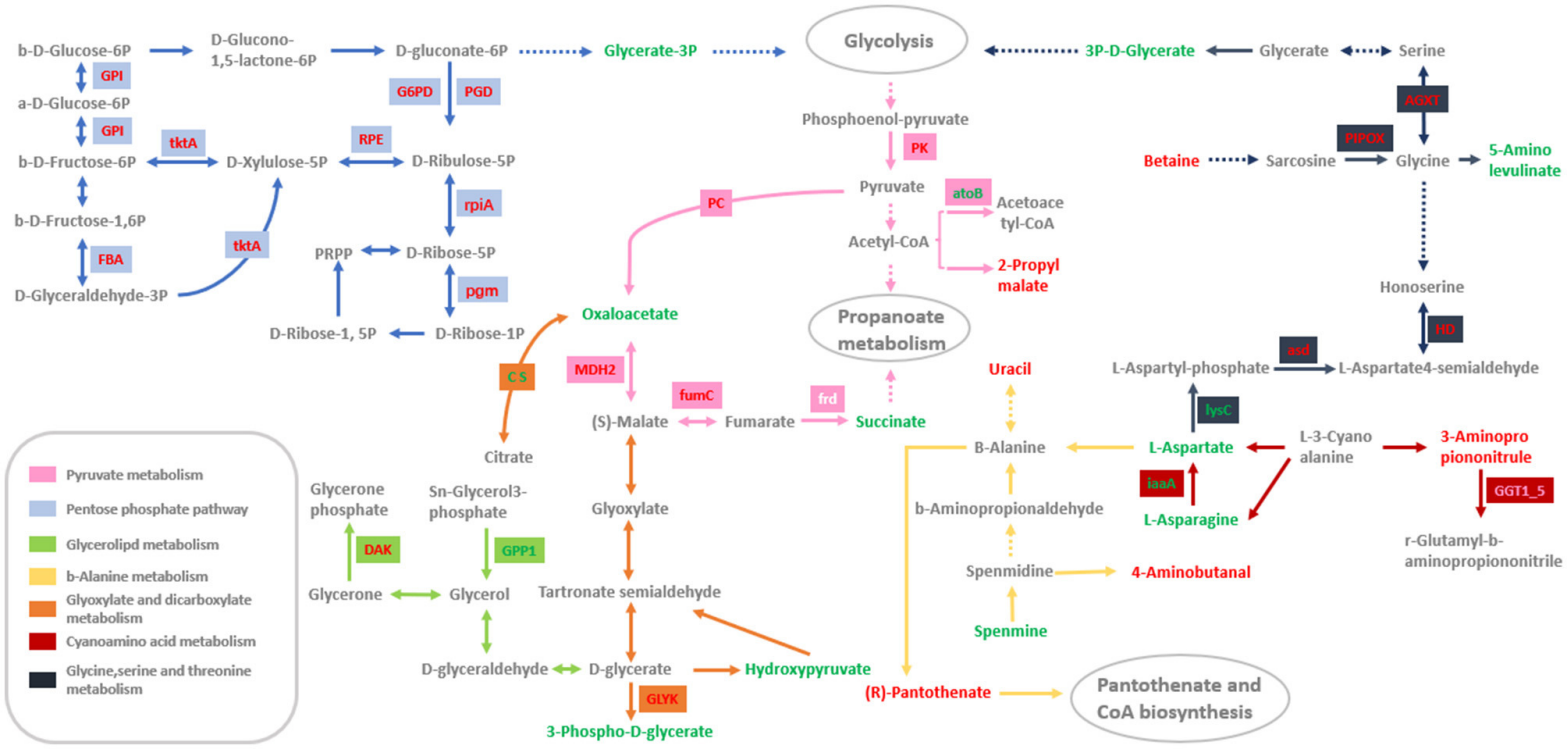
The seven pathways enriched by significant DEGs and DAMs involved in mycelium growth. The small square containing words represents the DEGs that participated in this pathway. The red word in the small square indicates that this gene is upregulated within the mycelium growth, while the green word indicates that this gene is downregulated. Similarly, the red words in each pathway indicate upregulated DAMs while the green words indicate downregulated.

The pentose phosphate pathway (PPP) exists commonly in a broad range of organisms and generates both NADPH and pentose molecules. It is an important pathway of sugar catabolism besides glycolysis (Tao et al., 2015). It begins with the glucose 6-phosphate molecules synthesized in the first reaction step of glycolysis, thus PPP competes for the use with glycolysis. Previous studies have shown that the pentose phosphate pathway is closely related to the growth and development of organisms and various environmental stresses (Esposito et al., 2003; Hou et al., 2007). The pentose phosphate pathway is one of the pathways commonly enriched by DEGs and DAMs in this study (Figure 6). In this pathway, the transketolase (tktA) encoding gene was downregulated during the mycelia development (Table 2). Transketolase catalyzes the transfer of a two-carbon ketol group from a ketose donor to an aldose acceptor; it requires thiamine pyrophosphate (TPP) as a cofactor and plays an important role in carbohydrate metabolism (Wilkinson and Dalby, 2019). The research found that with the overexpression of the transketolase gene (AO090023000345) in *Aspergillus oryzae*, the biomass of hyphae can increase 1.2-fold (Tamano and Miura, 2016). Moreover, the glucose-6-phosphate dehydrogenase (G6PDH) in this pathway was identified to be upregulated in the SD stage (Table 2). Feng et al. indicated that the enzymatic activity was significantly increased at the stage of fruiting body growth of *Pleurotus nebrodensis* (Feng et al., 2014). Meanwhile, Chen et al. found that the expression of glucose-6-phosphate dehydrogenase (G6PDH) was significantly higher in the *heterokarytic* mycelia than in the corresponding two homokarytic mycelia in *Volvaria volvacea*.

Fructose-diphosphate aldolase (Fba) is a soluble protein abundant in fungal cell walls and cytosol. It can catalyze the reversible reaction of fructose-1, 6-diphosphate cleavage into dihydroxyacetone phosphate and glyceraldehyde 3-phosphate, which is closely related to the energy metabolism of fungus (Rodaki et al., 2006; Lee et al., 2010). Studies have shown that the expression of Fba is significantly increased during the growth of *Candida albicans* hyphae, which indicates that Fba is related to the growth of *Candida albicans*. Ribose-5-phosphate isomerase (rpiA) catalyzes the interconversion of ribose-5-phosphate and ribulose-5-phosphate and maintains a balance between the ribose-5-phosphate and ribulose-5-phosphate in organisms (Zhang et al., 2003). This reaction ensures the conversion of sugar-phosphate to the intermediates of glycolysis and provides precursors for the synthesis of amino acids, vitamins, and nucleotides, which are essential for the growth and development of organisms (Hove-Jensen and Maigaard, 1993; Poulsen et al., 1999). Ribulose-phosphate 3-epimerase (RPE) converts monosaccharides including glucose into the nucleotide precursor of pentose sugars and plays a vital role in the development of a pool of NADPH (Guo et al., 2019). The research found that the disruption of RPE gene in *Escherichia coli* resulted in low cell growth (Shimaoka et al., 2005). Phosphoglucose isomerase (PGI) is involved in a variety of biological processes in organisms, such as supplying structural substances for cell wall synthesis pathways and participating in cell cycle regulation (Tuckman et al., 1997). The microorganisms will use other substances to synthesize glucose to maintain growth through the gluconeogenesis pathway when there is a lack of glucose and other hexoses in the environment, and phosphoglucose isomerase (PGI) is the key enzyme in the gluconeogenesis pathway (Tuckman et al., 1997). In this study, the genes of Fba, rpiA, RPE, and PGI participated in the pathway of pentose phosphate and were upregulated in the growth stage of SD (Table 2). In addition, Li et al. (2006) selected materials of *H. parviporum* at four development periods (18, 36, 72, and 120 h) to investigate gene expression patterns during the spore germination stages. The results showed that transcript levels of genes involved in glucose metabolism (phosphoglucomutase), amino acid metabolism (arginase, delta-1-pyrroline-5-carboxylate reductase, sulfur metabolism-negative regulator, imidazoleglycerol phosphate dehydratase), and protein synthesis (40S ribosomal protein S15) were significantly increased during the polarized growth (36 h) stage but decreased at early and late stages of mycelial growth (72–120 h) (Li et al., 2006). Phosphoglucomutase (pgm) was also identified in this pathway and the expression level of pgm was increased in the SD mycelium development stage (Table 2). These results suggested that the DEGs of tktA, G6PDH, Fba, rpiA, RPE, and PGI participated in the pentose phosphate pathway and may be related to mycelia development; further study on this is required.

Pyruvate is an intermediate metabolism production at the end of the glycolytic pathway, connected to hexose phosphate pathway (EMP) and tricarboxylic acid cycle (TCA) (Ogasawara et al., 2007). In our study, we detected DEGs and DAMs related to mycelia development, which were significantly enriched in the pathway of pyruvate metabolism. In this pathway, the succinate was less accumulated in the SM stage than in the SD stage (Table 2). Succinate is catalyzed by succinate dehydrogenase (frd). The research found that after 1 μg/mL penthiopyrad (succinate dehydrogenase inhibitor) treatment, mycelia of *S. sclerotiorum* strains showed increased apical branching and were denser compared with the control, and cell membrane permeability significantly increased (Mao et al., 2020). Although no significant reduction of succinate dehydrogenase expression was detected by the transcriptome data, we hypothesized that low succinate dehydrogenase expression results in less succinate accumulation, which promotes mycelium growth. Meanwhile, according to the DAMs and DEGs enrichment results, oxaloacetate and pyruvate carboxylase (PC) were also identified and enriched in the pyruvate metabolism pathway, and the expression level of pyruvate carboxylase was upregulated in the SD and SM stages in the compared groups HG-vs-SD and HG-vs-SM. Previous research reported that after overexpression of the PC gene (pyruvate carboxylase encoding gene) in *Pichia pastoris*, the content of oxaloacetate was increased by 109.6%, L-malic acid increased by 33.7%, and the cell biomass increased by 15.7%, which indicates that PC is propitious to the accumulation of oxaloacetate and L-malic acid and plays a significant role in promoting the growth of strain (Wu et al., 2011). Therefore, we also assume that the increased expression of pyruvate carboxylase may promote the accumulation of oxaloacetate and then promote the growth and development of mycelia during mycelial growth. Although the content of oxaloacetate is decreased within the mycelium growth in our study, we also assume that this metabolite is important to mycelium growth and it is worthy to study further.

Besides the above-mentioned DEGs and DAMs, we also identified a number of DEGs and DAMs that participated in pathways of cyanoamino acid metabolism (ko00460), glycine, serine, and threonine metabolism (ko00260), beta-alanine metabolism (ko00410), glycerolipid metabolism (ko00561), and glyoxylate and dicarboxylate metabolism (ko00630), which are considered as key genes and metabolites related to mycelium growth (Table 2). These genes and metabolites may also contribute to the growth and development of mycelia in *Morchella importuna*.

## 5. Conclusion

In the present study, we conducted comparative transcriptome analysis and metabolomic analysis to explore the key genes and metabolites involved in mycelium growth in *Morchella importuna*. As a result, 24 DEGs and 15 DAMs were identified by comparing the three growth stages of HG, SD, and SM. Most DEGs were genes related to carbohydrate metabolisms (“Starch and sucrose metabolism,” “Pentose and glucuronate interconversions,” “Tyrosine metabolism,” “Purine metabolism,” “Fructose and mannose metabolism,” and so on), such as *tktA, Fba, rpiA, RPE*, and *PGI*, whose expression was mostly activated at the SD stage. The accumulation of carbohydrates, such as glycerate-3P, oxaloacetate, and succinate, showed a downward trend in the development of *Morchella importuna* to promote mycelium growth. In the SD stage, the contents of cGMP and GMP were increased to regulate the synthesis of mycelium development-associated nucleotides, and secondary metabolites were accumulated to mediate ecological interactions and improve growth viability. All these results contribute to a better understanding of the molecular mechanisms of mycelium growth in *M. importuna* and provide new insights for optimizing the genetic breeding of *Morchella importuna*.

## Data availability statement

The datasets presented in this study can be found in online repositories. The names of the repository/repositories and accession number(s) can be found below: https://www.ncbi.nlm.nih.gov/, PRJNA882762.

## Author contributions

TF and JX: conceptualization. RR and ST: methodology. ST: software. TF, RR, ST, YZ, and YH: formal analysis. TF: investigation and writing—original draft preparation. TF ST, and JX: resources. MC and WZ: data curation. JX: writing—review and editing, visualization, supervision, and project administration. TF, JX, and ST: funding acquisition. All authors have read and agreed to the published version of the manuscript.
