# Supplementary material for: Transcriptomics combined with metabolomics unveiled the key genes and metabolites of mycelium growth in Morchella importuna
Source: Front Microbiol. 2023 Feb 1;14:1079353. doi: 10.3389/fmicb.2023.1079353 (PMC9929000; doi:10.3389/fmicb.2023.1079353)
Supplement: Supplementary file 5 [file Data_Sheet_1.docx]

Supplementary Material

# Supplementary Data

Supplementary dataset 1

Supplementary dataset 2

Supplementary dataset 3

Supplementary dataset 4

# Supplementary Figures and Tables

## Supplementary Figures

| **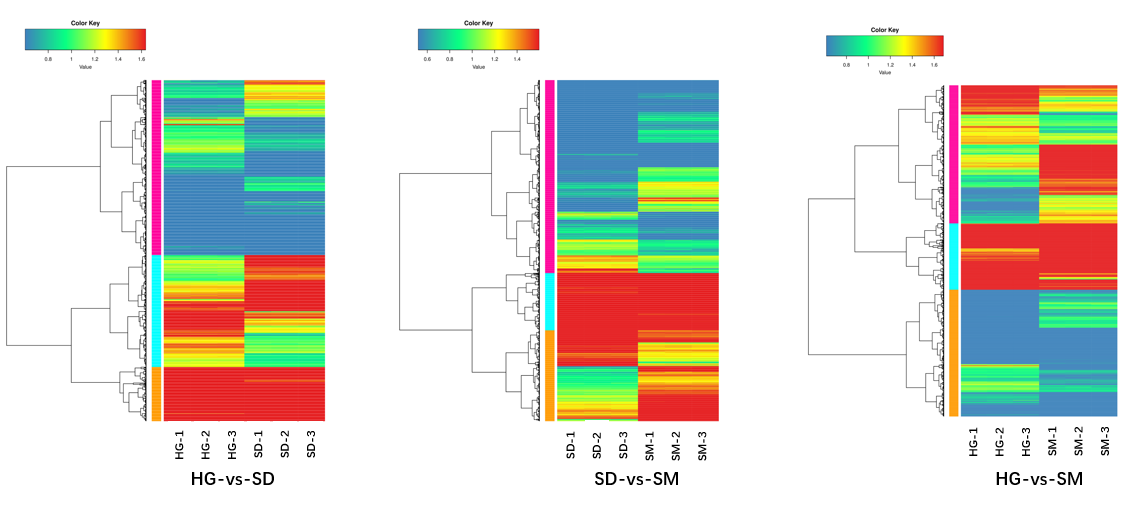** |
| --- |
| **Figure S1** Hierarchical clustering heatmap of DEGs at three compared groups. |

| 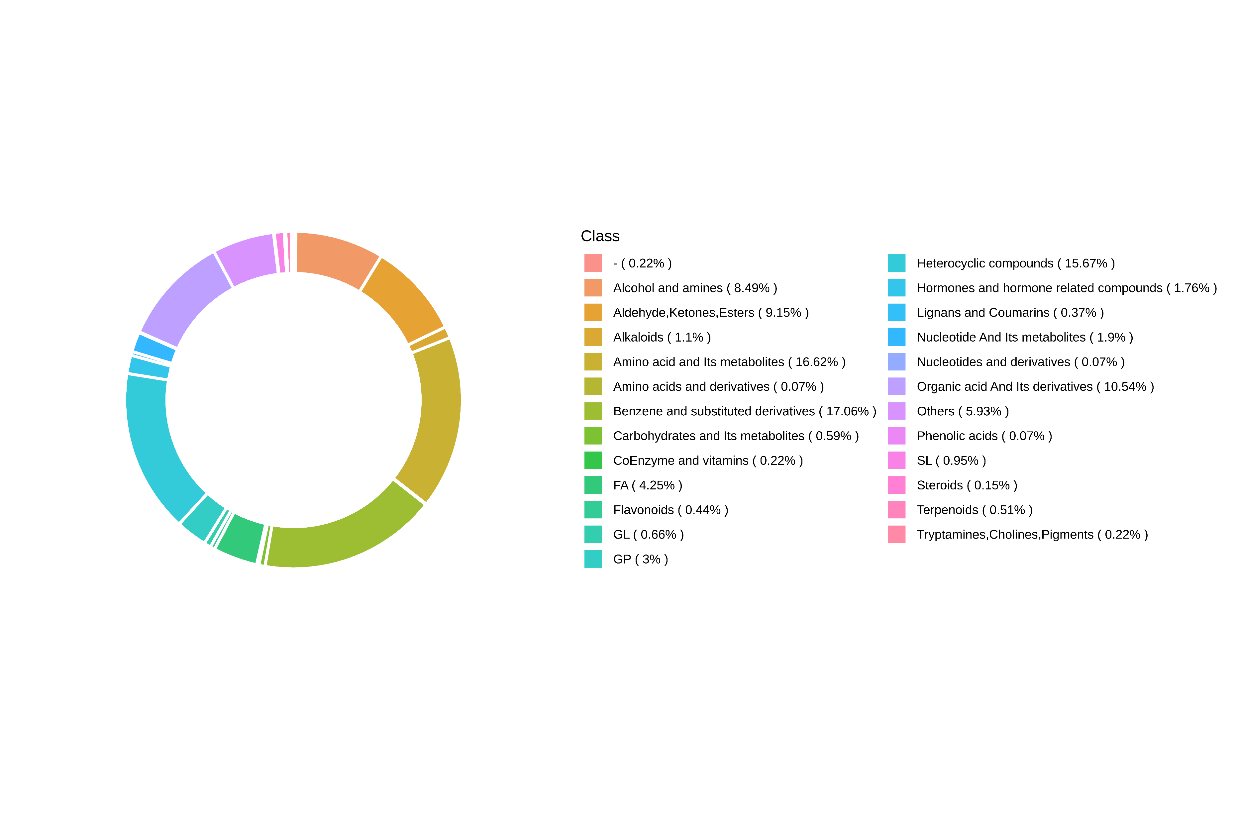 |
| --- |
| Positive ion mode |
| 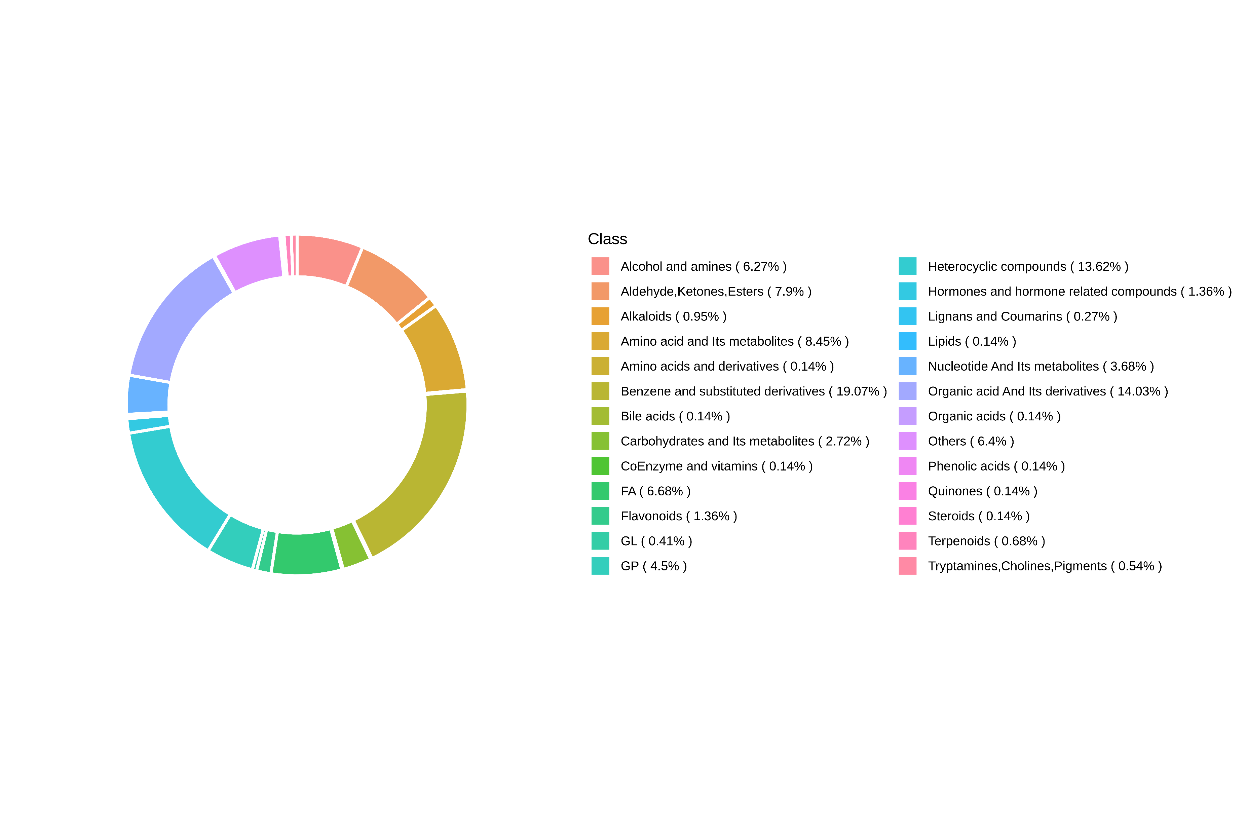 |
| Negative ion mode |
| **Figure S2.** The composition of metabolites identified by metabolomics under positive and negative ion mode. |

| 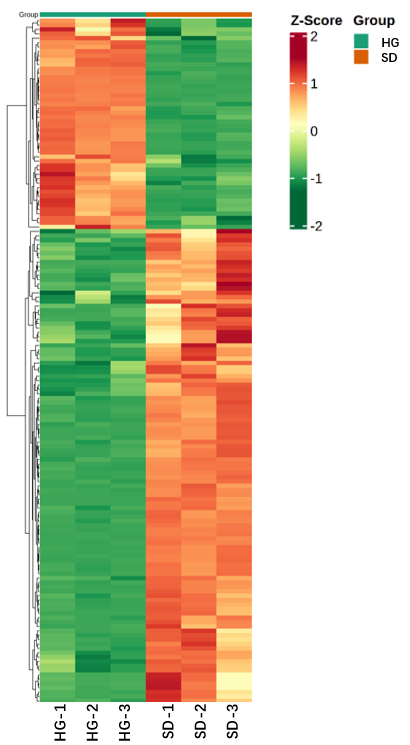 | 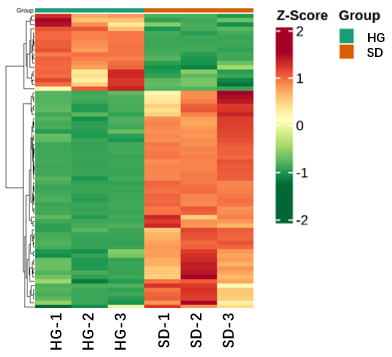 |
| --- | --- |
| Positive ion mode | Negative ion mode |
| HG-vs-SD | |
| 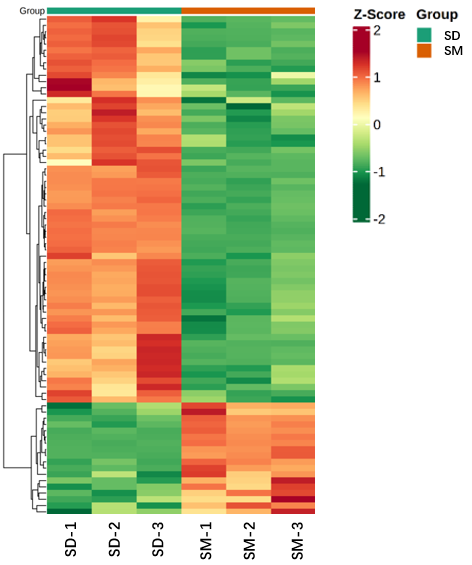 | 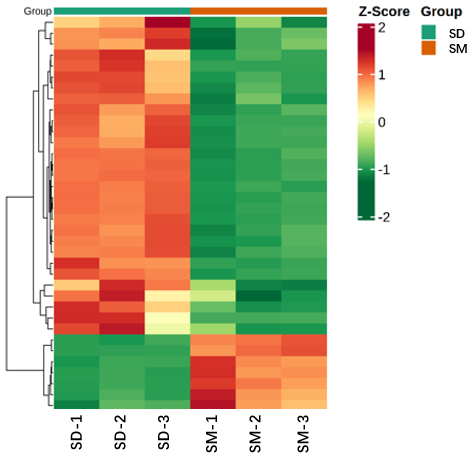 |
| Positive ion mode | Negative ion mode |
| SD-vs-SM | |
| 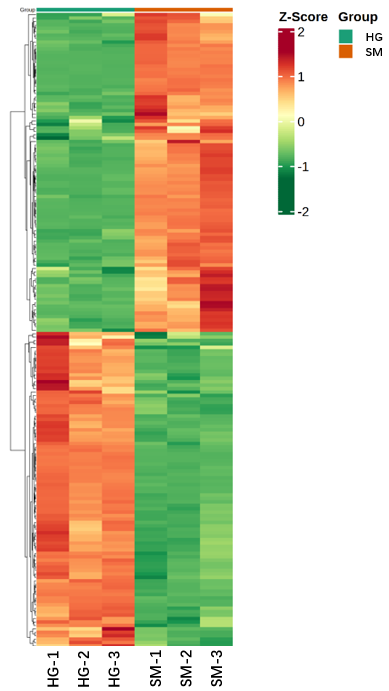 | 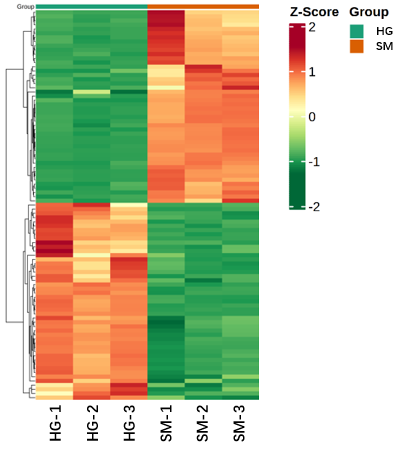 |
| Positive ion mode | Negative ion mode |
| HG-vs-SM | |
| **Figure S3.** Hierarchical clustering heatmap of DAMs at three compared groups under negative and negative ion mode.   \| 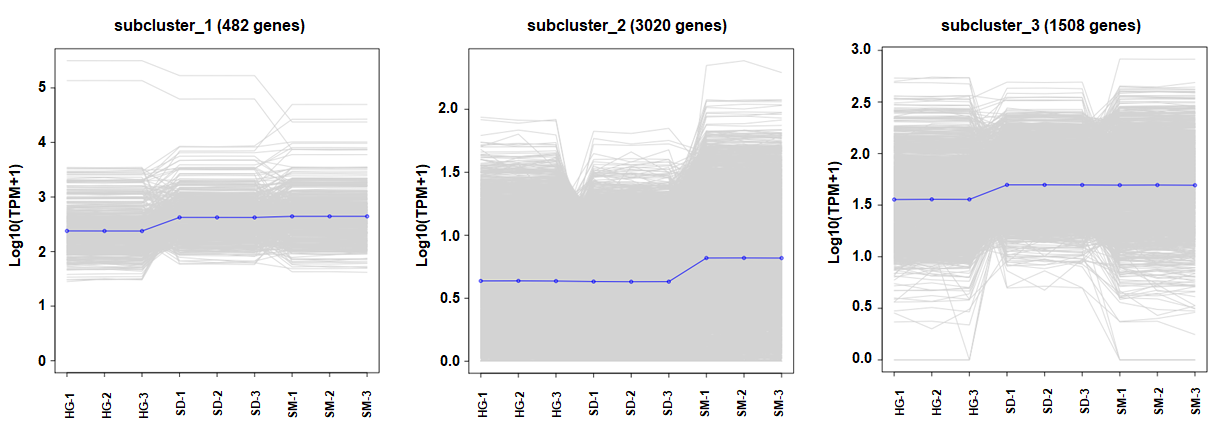 \| \| --- \| \| **Figure S4.** Expression modules of differentially expressed genes during the three growth stages of *Morchella importuna*. \|  \| 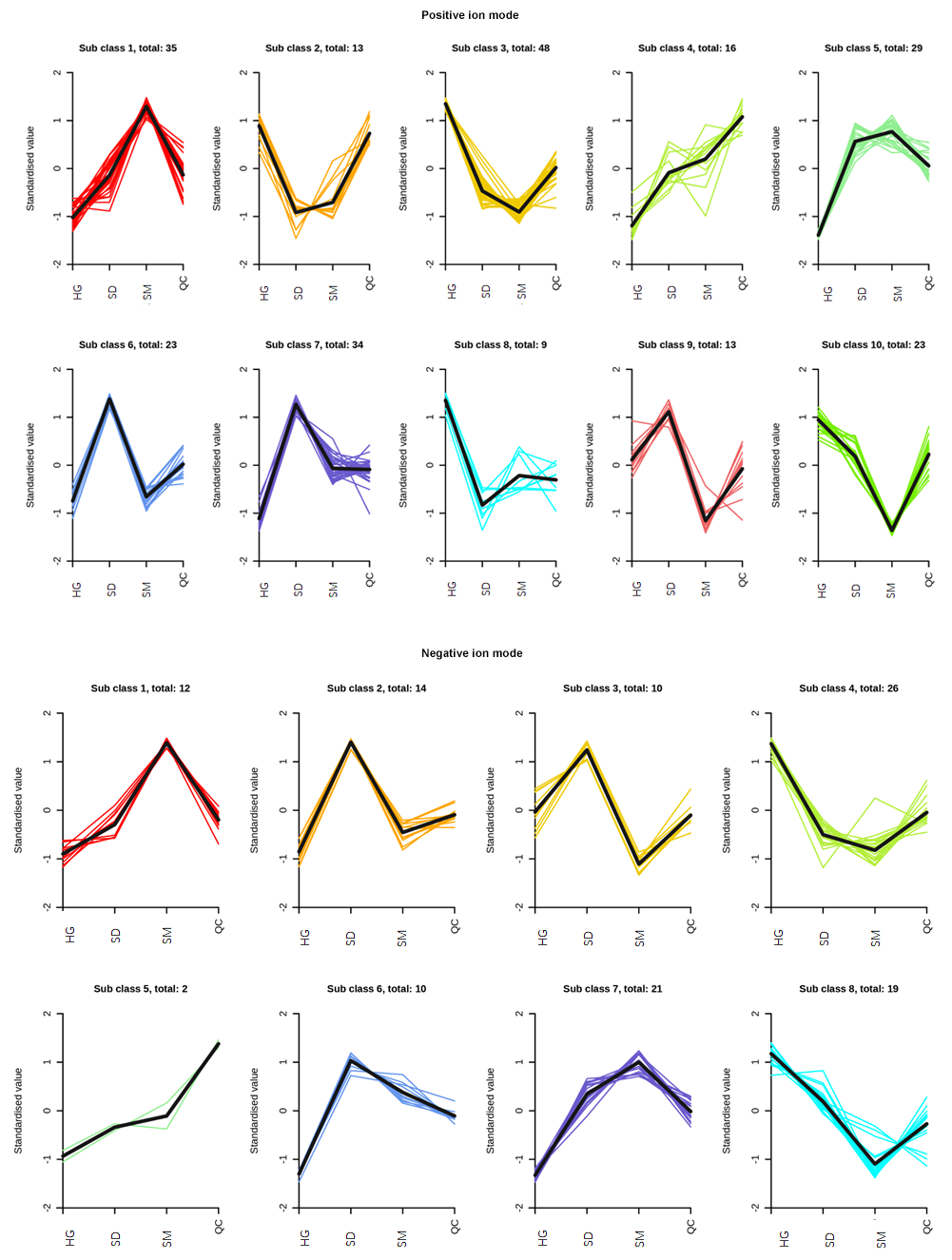 \| \| --- \| \| **Figure S5.** Dynamic metabolomics changes during the three growth stages of *Morchella importuna*. \| | |
| 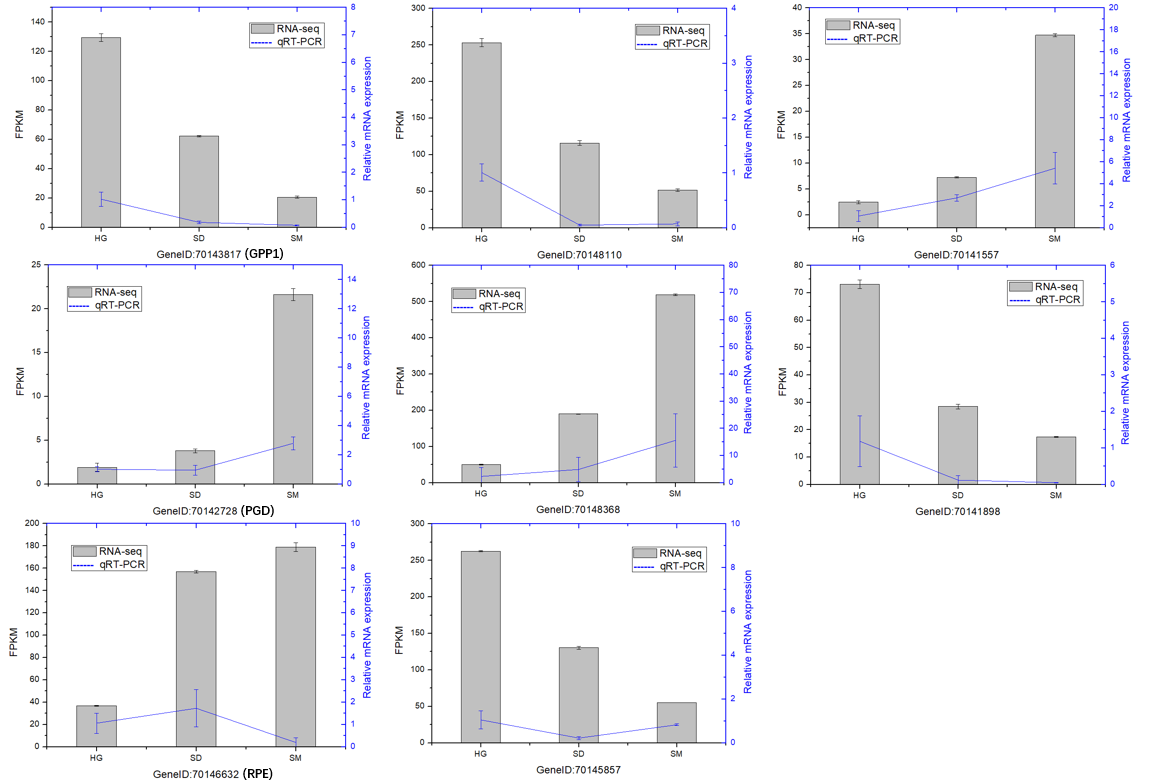 | |
|  | |
| **Figure S6**. The qRT-PCR verification diagram of eight DEGs. | |

## Supplementary Tables

**Table S1.** The nucleotide sequence of oligonucleotide primers of eight genes used in the present study for qRT-PCR experiment.

| **Gene ID** | **Primer name**  **(Sense/Anti-sense primer)** | **Nucleotide sequence** |
| --- | --- | --- |
| GeneID:70141557 | F | 5' CGGGTGTTGGTATGTGGG 3' |
|  | R | 5' CGGATTGACGGAAGTGTT 3' |
| GeneID:70141898 | F | 5' TACCCTGGCTACAGAATC 3' |
|  | R | 5' TCCAAGGTCCATTGAGTT 3' |
| GeneID:70142728 | F | 5' TCCGCACCCACCATCACA 3' |
|  | R | 5' CTCGCCAGGAACGCACAA 3' |
| GeneID:70143817 | F | 5' GCTGCTGACATCGCATACC 3' |
|  | R | 5' CTAATTCGCCAACTCCTTCG 3' |
| GeneID:70145857 | F | 5' CAGCCCAACAACGAGATT 3' |
|  | R | 5' CATTGACGAAACGGAAGG 3' |
| GeneID:70146632 | F | 5' CAGACTGGAGTAAGGGTGTT 3' |
|  | R | 5' CTGCGGCTTCATAGTGGA 3' |
| GeneID:70148110 | F | 5' TTGGCGTGTCAAACTTCGG 3' |
|  | R | 5' TGCCCTTGCTGGTGTTGT 3' |
| GeneID:70148368 | F | 5' CCTCCAACGACACCTTCCC 3' |
|  | R | 5' TGTAGCCGCTGAACTCCTG 3' |

**Table S2.** Summary of RNA-sequencing data.

| **Samples** | **Clean Read pairs** | **Q20(%)** | **Q30(%)** | **GC(%)** | **Total mapped reads (%)** | **Uniq mapped reads (%)** |
| --- | --- | --- | --- | --- | --- | --- |
| HG-1 | 20,044,040 | 98.8;97.56 | 95.94;93.02 | 50.69;50.01 | 18,881,149  (94.20%) | 10,876,309  (54.26%) |
| HG-2 | 21,171,112 | 98.8;97.56 | 95.94;93.02 | 50.69;50.01 | 19,943,203  (94.20%) | 11,384,614  (53.77%) |
| HG-3 | 20,185,124 | 98.8;97.56 | 95.94;93.02 | 50.69;50.01 | 19,011,634  (94.19%) | 10,809,128  (53.55%) |
| SD-1 | 20,850,044 | 98.85;97.56 | 96.06;92.84 | 50.63;50.22 | 19,699,503  (94.48%) | 14,281,307  (68.50%) |
| SD-2 | 20,145,076 | 98.85;97.56 | 96.07;92.84 | 50.63;50.22 | 19,031,431  (94.47%) | 13,825,280  (68.63%) |
| SD-3 | 21,138,372 | 98.85;97.56 | 96.07;92.84 | 50.63;50.22 | 19,980,210  (94.52%) | 14,437,958  (68.30%) |
| SM-1 | 20,865,284 | 98.82;97.52 | 96.01;92.89 | 49.72;49.54 | 19,860,662  (95.19%) | 18,863,132  (90.40%) |
| SM-2 | 20,316,336 | 98.82;97.52 | 96.01;92.89 | 49.72;49.52 | 19,339,335  (95.19%) | 18,371,211  (90.43%) |
| SM-3 | 20,393,984 | 98.82;97.52 | 96;92.89 | 49.72;49.52 | 19,413,741  (95.19%) | 18,433,030  (90.38%) |

**Table S3.** The statistic of DEGs in three compared groups.

| **Compared group** | **Total** | **Up** | **Down** |
| --- | --- | --- | --- |
| HG-vs-SD | 2,648 | 1,337 | 1,311 |
| SD-vs-SM | 2,368 | 1,331 | 1,037 |
| HG-vs-SM | 3,650 | 1,977 | 1,673 |

**Table S4.** The statistic of metabolites in positive and negative ion mode.

|  | **All** | **Positive ion mode** | **Negative ion mode** |
| --- | --- | --- | --- |
| Metabolites counts | 2100 | 1,366 | 734 |
| Secondary metabolites count | 916 | 634 | 282 |

**Table S5.** The statistic of significant DAMs in three compared groups under positive and negative ion mode.

| **Ion mode** | **Compared group** | **All significant DAMs** | **Up regulated** | **Down regulated** |
| --- | --- | --- | --- | --- |
| Positive ion mode | HG-vs-SD | 156 | 108 | 48 |
|  | SD-vs-SM | 80 | 18 | 62 |
|  | HG-vs-SM | 185 | 93 | 92 |
| Negative ion mode | HG-vs-SD | 69 | 51 | 18 |
|  | SD-vs-SM | 36 | 7 | 29 |
|  | HG-vs-SM | 93 | 46 | 47 |
